# The Effect of Dehydroepiandrosterone Treatment on Neurogenesis, Astrogliosis and Long-Term Cocaine-Seeking Behavior in a Cocaine Self-Administration Model in Rats

**DOI:** 10.3389/fnins.2021.773197

**Published:** 2021-11-26

**Authors:** Hadas Ahdoot-Levi, Ofri Croitoru, Tzofnat Bareli, Einav Sudai, Hilla Peér-Nissan, Avi Jacob, Iris Gispan, Rachel Maayan, Abraham Weizman, Gal Yadid

**Affiliations:** ^1^Neuropharmacology Laboratory, The Mina and Everard Goodman Faculty of Life Sciences, Ramat-Gan, Israel; ^2^The Leslie and Susan Gonda (Goldschmied) Multidisciplinary Brain Research Center, Ramat-Gan, Israel; ^3^The Laboratory of Biological Psychiatry, Felsenstein Medical Research Center and Sackler Faculty of Medicine, Tel Aviv University, Petah Tikva, Israel; ^4^Research Unit, Geha Mental Health Center, Petah Tikva, Israel

**Keywords:** addiction, neurogensis, astrogliosis, cocaine, self-administration, DHEA

## Abstract

Cocaine addiction is an acquired behavioral state developed in vulnerable individuals after cocaine exposure. It is characterized by compulsive drug-seeking and high vulnerability to relapse even after prolonged abstinence, associated with decreased neurogenesis in the hippocampus. This addictive state is hypothesized to be a form of “memory disease” in which the drug exploits the physiological neuroplasticity mechanisms that mediate regular learning and memory processes. Therefore, a major focus of the field has been to identify the cocaine-induced neuroadaptations occurring in the usurped brain’s reward circuit. The neurosteroid dehydroepiandrosterone (DHEA) affects brain cell morphology, differentiation, neurotransmission, and memory. It also reduces drug-seeking behavior in an animal model of cocaine self-administration. Here, we examined the long-lasting effects of DHEA treatment on the attenuation of cocaine-seeking behavior. We also examined its short- and long-term influence on hippocampal cells architecture (neurons and astrocytes). Using a behavioral examination, immunohistochemical staining, and diffusion tensor imaging, we found an immediate effect on tissue density and activation of astrocytes, which has a continuous beneficial effect on neurogenesis and tissue organization. This research emphasizes the requites concert between astrocytes and neurons in the rehabilitation from addiction behavior. Thus, DHEA may serve as a treatment that corrects brain damage following exposure to and abstinence from cocaine.

## Introduction

Drug addiction is a complex disease involving mind and body, defined as the compulsion to consume a drug while losing control over the amount consumed (Everitt et al., 2001). Cocaine usage disorder is characterized by a high propensity for repeated drug use and addictive substance relapse, which can occur during short periods of withdrawal or even following years of successful abstinence (Rounsaville et al., 1991; Ciccarone, 2011). Therefore, the two main challenges associated with treating cocaine dependence are treatment retention and relapse (Penberthy et al., 2010).

The neuroadaptations following cocaine administration have been studied extensively to determine the consequences of cocaine abuse and potential addiction treatment targets. Merging reports emphasize the importance of both the glutamatergic signaling-pathway and the learning and memory circuit for switching from substance use- to substance abuse-behavior (Doyle et al., 2014). Substance-related memories, the shift to addiction (Meyers et al., 2006) and relapse, transpire in the hippocampus (among other brain regions), a part of the mesolimbic system (Shen et al., 2006; Hernández-Rabaza et al., 2008). Hippocampal neurogenesis is at the core of hippocampal plasticity: the newly formed neurons in the dentate gyrus (DG) integrate into the existing neural network and contribute to hippocampal function. Ample evidence shows neurogenesis reduction in response to various addictive drugs, such as cocaine (Sudai et al., 2011), alcohol (Herrera et al., 2003), opiates (Teuchert-Noodt et al., 2000), and amphetamines (Eisch et al., 2000; Domínguez-Escribà et al., 2006; Eisch and Harburg, 2006). Also, preventing neurogenesis prior to drug exposure increases susceptibility to drug-seeking behavior (Noonan et al., 2010).

Dehydroepiandrosterone is a neuroprotective steroid that modulates neuronal function, stimulates neurogenesis in the hippocampus of rats, promotes survival of newly formed neurons, and prevents corticosterone-induced suppression (Karishma and Herbert, 2002). DHEA and its sulfate ester, DHEA-S, represent the most abundant steroid hormones in the body. In humans, levels of DHEA and DHEA-S are altered during addiction to substances of abuse (Buydens-Branchey et al., 2002). Specifically, significantly lower levels of DHEA-S were observed in abusers that relapsed (Shoptaw et al., 2004). In a clinical trial conducted in a therapeutic center where addicts consumed add-on DHEA (100 mg/day) or placebo daily for at least 30 days during their routine treatment, DHEA raised the probability to successful remission from drug usage (Ohana et al., 2016). Controlled experimental studies using animal models confirmed the clinical observations (Doron et al., 2006; Maayan et al., 2006; Yadid Gal et al., 2010) regarding the effect of DHEA on drug-seeking behaviors at different phases of the self-administration model: acquisition, maintenance, and reinstatement in the short-term (immediately after treatment during the extinction phase). In addition, it has been shown that DHEA treatment does not disrupt general operant behavior by testing its effect on sucrose intake (Doron et al., 2006).

This study aimed to examine post-DHEA treatment’s long-term (one month) effect on drug-seeking behavior and neurogenesis in rats. We used diffusion tensor imaging (DTI), a non-invasive method, to evaluate longitudinal changes of hippocampus tissue in a month-long period of DHEA treatment during withdrawal from repeated cocaine self-administration. We examined proliferation in the short-term and then neurogenesis concurrently with the DHEA effect on drug-seeking behavior one month post-treatment.

Since preliminary data of DTI showed dynamic changes in tissue density during abstinence and after DHEA treatment, which were inversely proportional with proliferation and neurogenesis changes, we further sought factors other than the neurogenesis that could affect the DTI results. Although most literature on substance use disorder concentrates on the intactness and function of neurons, emerging evidence indicates astroglia as a key element in neuropathology (Verkhratsky et al., 2016). Astrocytes play a critical role in neuromodulation, neuroprotection, axon guidance control during development, and homeostasis preservation in the CNS (Kimelberg and Norenberg, 1989). Also, astrocytes regulate glutamatergic synaptic plasticity by controlling the extracellular glutamate concentration *via* coordinated uptake and release, hence contributing to learning and memory (Scofield and Kalivas, 2014).

Interestingly, DHEA was shown to have neuroactive effects on astroglial function in different experimental models (Arbo et al., 2016). Moreover, DHEA is a stimulant of anti-inflammatory processes in astrocytes (Buyanova et al., 2017).

Therefore, in the current study, we extended our examination of the effects of DHEA treatment also to astrocytes and related it to neurogenesis.

## Materials and Methods

### Subjects

Total 176 Male Sprague–Dawley rats (250–280 g) were used. Rats were purchased from Envigo Inc., were maintained (in pairs) on a 12–12 h light-dark cycle (lights off at 07:00 AM) with free access to food and water. All experimental procedures were approved by the Animal Care and Use Committee of Bar Ilan University and performed in accordance with the guidelines of the National Institute of Health.

### Cocaine Self-Administration

Rats were trained to self-administer cocaine (National Institute on Drug Abuse, Baltimore, MD, United States) for 16 days under an FR-1 schedule of reinforcement. 5 days after catheterization, rats were transferred to operant conditioning chambers (Med-Associates, Inc.; St. Albans, VT) for one hour daily during their dark cycle. Each self-administration chamber (30⋅25⋅22 cm) had two levers, active and inactive, located 9 cm above the chamber’s floor. The self-administration chambers and the computer interface were built locally and controlled by a computer program written by Steve Cabilio (Concordia University, Montreal, PQ, Canada).

An active lever press generated a cocaine infusion (1.5 mg/kg, 0.13 ml, 20 s/infusion; cocaine was obtained from the National Institutes of Drug Abuse, North Bethesda, MD) through the IV catheter, which was connected to an infusion pump. Throughout cocaine infusion intervals, a light located above the active lever was lit for 20 s. During the 20-s intervals, active lever presses were recorded, but no additional cocaine reinforcement was provided. Presses on the inactive lever did not activate the infusion pump and light. The number of active lever responses, infusions, and inactive lever responses was recorded. Rats were returned to their home cages at the end of the daily session.

### Cocaine Extinction and Reinstatement to Cocaine

Rats subjected to DHEA (COC-DHEA group) or vehicle (COC-SAL group) injections were placed daily in the operant chamber for one hour without receiving cocaine in response to active lever pressing, until lever-pressing behavior resembled extinguished values. When the rats’ pressing on the active lever decreased by less than 80% deviation from the first day of extinction, it was assumed to be non-reinforced by cocaine. The control groups were trained to self-administer saline, subjected to DHEA (SAL-DHEA group) or vehicle (SAL-SAL group) during extinction. For the next 28 days, rats were not exposed to cocaine or operant chambers. After a total of 36 days since their last exposure to cocaine, rats received a priming injection of cocaine (10 mg/kg, i.p.) before placement in the self-administration chambers (operant chamber). Active lever responses, reinforcements, and inactive lever responses were recorded.

### Dehydroepiandrosterone Administration

Dehydroepiandrosterone (Sigma Chemicals; St Louis, MO) was dissolved in 0.3 ml of dimethylsulfoxide (DMSO) and then diluted to 1 mg/ml of saline (final DMSO concentration was 1%). Rats were injected i.p. with DHEA (2 mg/kg) or vehicle (saline containing 1% of DMSO) 90 min prior to exposure to the operant chamber. Based on previous research showing that cocaine addicts that had a twofold increase in plasma DHEA-S levels compared to other addicts were less prone to relapse and based on prior evaluation of the effect of DHEA injections on DHEA-S plasma levels in rats, we found that the optimal DHEA dose to achieve a twofold increase in DHEA-S plasma levels was 2 mg/kg in cocaine self-administrating rats (Everitt et al., 2001).

### BrdU Administration

Rats were housed under standard conditions and injected i.p. with BrdU (Sigma-Aldrich; 50 mg per kg body weight) 3 injections at 4-h intervals for one day after reaching stable maintenance levels. They continued to live in this environment for 24 h or 28 days after the last injection. Then, they were euthanized and perfused transcranial, first with PBS and then with 4% paraformaldehyde.

### Tissue Preparations

Phosphate buffer (PBS) perfusion was performed to the left ventricle in the rat heart until the blood washed out through the right atrium, then 4% paraformaldehyde was injected in the same manner for brain fixation. Brains were removed, post-fixed overnight, and equilibrated in phosphate-buffered 30% sucrose. Free-floating, 40-μm-thick coronal hippocampal sections were collected on a freezing cryostat and stored at 4°C before immunohistochemistry.

### Immunohistochemistry

For BrdU staining of rat specimens, tissue sections were washed with PBS, incubated in 2N HCl at 37°C for 30 min, and then blocked for one h with blocking solution (PBS containing 20% normal horse serum and 0.5% Triton X-100). The tissue sections were stained overnight with specified combinations of the following primary antibodies: rat anti-BrdU (1:200; Oxford Biotechnology, Kidlington, Oxfordshire, United Kingdom) and mouse anti-NeuN (1:200; Chemicon, Temecula, CA). Secondary antibodies used for both rat tissues were Cy-3-conjugated donkey anti-rat (1:200; Jackson ImmunoResearch, West Grove, PA) and Cy-2 donkey anti-mouse (1:200; Jackson ImmunoResearch, West Grove, PA).

Astrocytes were immunohistochemically stained for S100β. Tissue sections were rinsed in PBSx1 + 0.1% triton and then blocked in 20% normal horse serum + 0.1% triton + PBSx1. s100β antibody (1: 7,500; 16H24L21, Invitrogen) with PBSx1 + 2% normal horse serum + 0.1% triton solution. The slices were stored at 4°C overnight. The second (fluorescent) 488 alexa antibody (green) was introduced at a ratio of 1: 1000 (A11034, Invitrogen) in PBSx1 + 0.1% triton solution for one hour. Slices were washed again and stained with fluorescent nucleus by DAPI (1: 10,000; F6057, Sigma) in PBSx1 + 0.1% triton solution for 10 min.

For microscopic analysis, we used a fluorescent microscope (40× magnifications). Neurogenesis in the dentate gyrus was evaluated by counting the cells that were double-labeled with BrdU and NeuN. We counted the number of labeled cells in nine coronal sections (360 μm apart) per rat brain, stained them, and mounted them on coded slides. To obtain an estimate of the total number of labeled cells per dentate gyrus, we multiplied the total number of cells counted in the selected coronal sections from each brain by the volume index (the ratio between the volume of the dentate gyrus and the total combined volume of the selected sections).

For astrocyte analysis, we used a Leica SP8 confocal microscope; excitation wavelengths: 488 nm, 405 nm, using a white-light laser. A 3D image of the ventral and dorsal dentate gyrus was performed; Scale bar is 50 μm. All raw images were analyzed using Leica’s analysis software.

### Intravenous Catheterization

The rats were implanted with intravenous Silastic catheters (Dow Corning, Midland, MI) into the right jugular vein (Ciccarone, 2011). The catheter was secured to the vein with silk sutures and was passed subcutaneously to the top of the skull, where it exited into a connector (a modified 22-gage cannula; Plastics One, Roanoke, VA) mounted to the skull with MX-80 screws (Small Parts, Inc., Miami Lakes, FL) and dental cement (Yates and Bird, Chicago, IL). Catheter patency was tested daily by injecting sterile saline.

### Diffusion Tensor Imaging

Diffusion-tensor imaging (DTI) is a technique in which diffusion is measured in a series of different spatial directions. The shape and orientation of the diffusion tensor are determined at each pixel.

MRI was performed at Tel-Aviv University using a 7T MRI scanner (Bruker, Karlsruhe, Germany) with a 30-cm bore and a gradient strength of up to 400 mT/m. The MRI protocol included diffusion tensor imaging (DTI) acquisition with a diffusion-weighted (DW) spin-echo echo-planar-imaging (EPI) pulse sequence with the following parameters: TR/TE = 4000/25 ms, Δ/δ = 10/4.5 ms, four EPI segments, and 15 non-collinear gradient directions with a single *b*-value shell at 1000 s/mm^2^; and one image with a *b*-value of 0 s/mm^2^ (referred to as *b*0). Geometrical parameters were: 20 slices, each 0.48 mm thick (brain volume) and with in-plane resolution of 0.2 × 0.2 mm^2^ (matrix size 128 × 128; FOV 25.6 mm^2^). The imaging protocol was repeated three times for signal averaging and compensated for acquisition in which significant head motion was observed. Each DTI acquisition lasted 4.5 min, and the entire MRI protocol lasted about 20 min.

### MRI Image Analysis

Image analysis included DTI analysis of the DW-EPI images to produce the fractional anisotropy (FA), apparent diffusion coefficient (ADC), and radial and axial diffusivity indexed maps (RD). DTI enables quantifying the motional anisotropy of water molecules and can provide images with high contrast toward the white matter. Lower FA values may indicate lower tissue directionality and organization or reduced axonal and myelin density. Higher ADC values may indicate reduced tissue density and integrity.

The DTI analysis was implemented in Matlab (©Mathworks, United States) using in-house software (Schnable et al., 2009). For statistical comparisons between rats, each rat brain volume was normalized with a template rat atlas allowing voxel-based statistics. All image transformations and statistical analyses described below were carried out using SPM2 software (Wellcome Trust Centre for Neuroimaging, London, United Kingdom). Each rat data set was normalized to the template images (registered to a digitized version of Paxinos Rat Brain Atlas (Nie et al., 2013). The normalization procedure included four steps. (a) All *b*0 images initially underwent bias correction. (b) The *b*0 image for each rat was co-registered with the *b*0 template (using a 6-parameter rigid-body transformation). The co-registration parameters were then applied to the different DTI indexed maps (FA, ADC, and radial and axial diffusivities). (c) The registered FA maps were normalized to the FA template (having a spatial resolution of 0.066 × 0.066 × 0.48 mm^3^) using a 12-parameter affine non-linear transformation and 0.2 mm smoothing (which was used only for the parameter estimation procedure and not smoothing of the output image). The normalization parameters were then applied on all DTI indexed maps, including the FA, ADC, and radial and axial diffusivities. (d) The normalized indexed maps were smoothed with a 0.3-mm Gaussian kernel.

### Statistical Analysis

For the behavioral data (cocaine self-administration, extinction, and reinstatement test), we applied one-, two-, or three-way ANOVA with or without repeated measures, followed by Bonferroni’s multiple comparisons test. The histological data (cell proliferation and survival) were analyzed by one-way ANOVA, followed by Bonferroni’s multiple comparisons test. DTI data were analyzed by one-way ANOVA with or without repeated measures, followed by Bonferroni’s multiple comparisons test, or a two-tailed Student’s unpaired *t*-test was used as appropriate. Data are presented as mean ± SEM. Results were considered significantly different if *P* < 0.05.

## Results

### Dehydroepiandrosterone Effect on Long-Term Reinstatement

**FIGURE 1:**
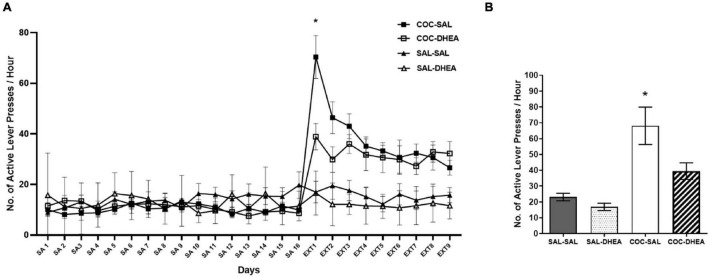
Self-administration and reinstatement test. **(A)** Effect of DHEA on cocaine-seeking during extinction. Rats (*n* = 32) were trained for cocaine self-administration (SA), days 1–16. A three-way ANOVA with repeated measures (drug × treatment × days) revealed a significant three-way interaction [*F*(24,627) = 2.057, *p* < 0.01]. This was followed up by a two-way ANOVA on the data from Ext1, which also revealed a significant interaction [*F*(24,260) = 2.024]. A significant number of active lever-presses on the first day of extinction in the COC-SAL group (*n* = 8) compared to the COC-DHEA group (*n* = 8). SAL-SAL (*n* = 8) and SAL-DHEA (*n* = 8) groups did not demonstrate any change in active lever presses at Ext 1 (*post hoc* *Bonferroni test, *p* < 0.05). **(B)** Reinstatement test: number of active lever-presses for 1 h, one month after the end of the extinction phase. COC-SAL and COC-DHEA groups were injected with cocaine (10 mg/kg. i.p.) and put in the operant chambers. The SAL-SAL group received a saline injection. There was a significant effect [*F*(3,28) = 11.82, *p* < 0.0001] of DHEA treatment during drug extinction on drug-seeking behavior in the reinstatement test (*Bonferroni tests, *p* < 0.05).

To examine long-term effects of DHEA treatment, we trained rats daily for one hour of cocaine self-administration until reaching constant drug consumption levels (Figure 1A). After reaching maintenance, the rats underwent an extinction phase: they were treated with DHEA (COC-DHEA group) or saline (COC-SAL group) and placed in the operant chamber for one hour a day without cocaine reinforcement. For the reinstatement test (36 days since last exposure to cocaine), we gave rats a priming injection of cocaine (10 mg/kg, i.p.), then put them in the operant chamber and measured their number of active lever-presses. The control group SAL-DHEA was trained with saline vehicles throughout maintenance and treated with DHEA throughout extinction. The SAL-SAL group was treated with saline vehicles during the whole procedure. Results show that the COC-SAL group pressed significantly more on the active lever than the COC-DHEA group from the first day of DHEA treatment (Ext1 Figure 1A), indicating higher craving for the drug. In the reinstatement test, the DHEA treatment shows a long-term effect, with significantly less active lever presses for the COC-DHEA group than the COC-SAL group (Figure 1B).

### The Effect of Dehydroepiandrosterone Treatment on Proliferation and Neurogenesis

**FIGURE 2:**
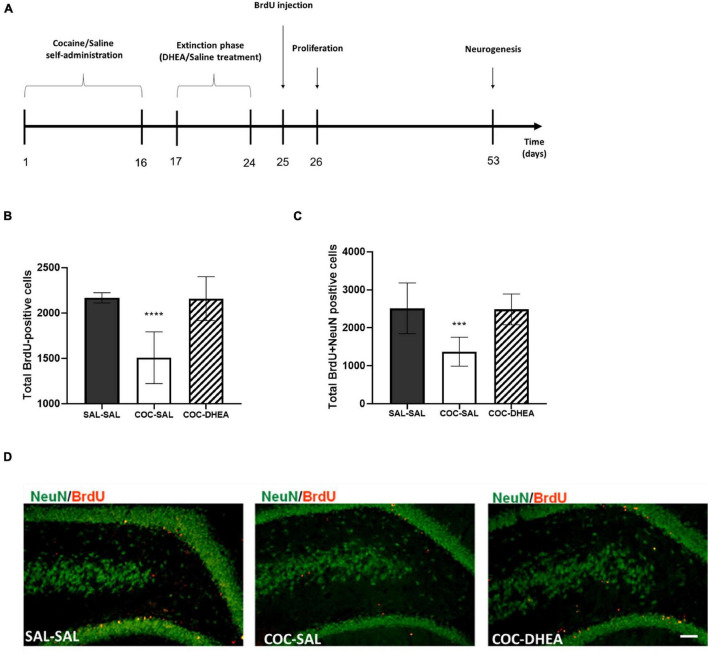
The effect of chronic DHEA treatment on hippocampal proliferation and neurogenesis of cocaine self-administrated rats. **(A)** Flow chart of proliferation and neurogenesis experiment procedures. 0–16 days: self-administration training of cocaine or saline. 17–24 days: extinction phase and DHEA or Saline treatment. 24 h or 28 days after BrdU injection, brains were excised for proliferation or neurogenesis measurements. **(B)** Proliferation quantification: BrdU positive cells in the dorsal DG (*n* = 7–10). **(C)** Neurogenesis quantification: newly generated neurons NeuN and BrdU positive (*n* = 10 per group). One-way ANOVA of the number of newly formed cells [proliferation; *F*(2,21) = 23.91, *p* < 0.0001] and mature neurons [neurogenesis; *F*(2,27) = 16.82, *p* < 0.0001] revealed a significant main effect. A *post hoc* Bonferroni test showed significance between COC-DHEA vs. COC-SAL groups, and SAL-SAL vs. COC-SAL groups (**** *p* < 0.0001, *** *p* < 0.001 Bonferroni’s multiple comparison test). Diversely, COC-DHEA vs. SAL-SAL groups, showed no significant main effect. **(D)** Representative micrographs of newly generated neurons: NeuN (green) and BrdU (red) positive in the dorsal DG, 40× magnification.

Previous research in our lab found that high doses of cocaine (1.5 mg/kg) self-administration reduce proliferation (newly formed cells) and neurogenesis (newly formed cells that mature to neurons) (Rounsaville et al., 1991). Since DHEA showed a significant effect on the behavioral aspect, we decided to examine whether treatment with DHEA can alter the proliferation and neurogenesis decrease—a process that indicates the long-term effect of treatment. We conducted the experiment according to the flow chart in Figure 2A. For proliferation examination, brains were excised 24 h after the BrdU injection. For neurogenesis measurements, brains were excised after 28 days.

Significantly more newly formed cells (cells positive for BrdU Figure 2B) and neurons (cells positive for BrdU and NeuN Figures 2C,D) were found in the dentate gyrus of the COC-DHEA than of the COC-SAL rats. No difference was found between the COC-DHEA and SAL-SAL groups, indicating a normalization of proliferation and neurogenesis after DHEA treatment. Noteworthy, DHEA administration at this dosage does not affect newly formed cells or neurons in the dentate gyrus of naïve rats (Supplementary Figure 1B).

### The Effect of Dehydroepiandrosterone Treatment on Diffusion Tensor Imaging Parameters

**FIGURE 3:**
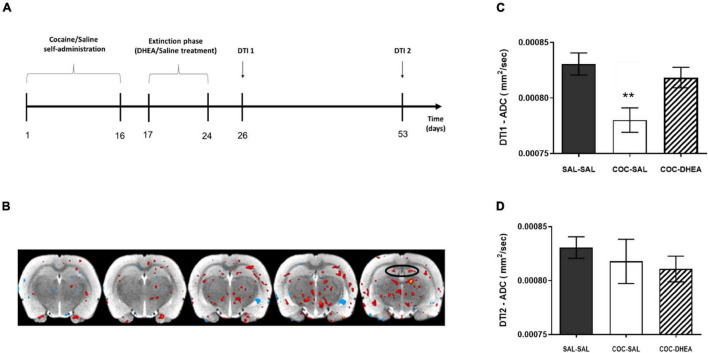
Effect of DHEA treatment on average diffusion in dentate gyrus area after cocaine self-administration. **(A)** Flow chart of DTI experimental procedures: rats were trained to self-administer cocaine or saline for 16 days. After rats achieved stable maintenance, they were treated with DHEA or saline during 8 days of extinction. 48 h later, a part of the rats was DTI scanned, corresponding to proliferation measurement (DTI1). A second group underwent the DTI scan after 28 days, corresponding to neurogenesis measurement (DTI2). **(B)** An illustrative image of the comparison between ADC values in COC-SAL vs. SAL-SAL brains. Red stain indicates a decrease in ADC value; blue indicates an increase in ADC value. **(C,D)** Quantification of the average diffusion in the tissue, ADC parameter, in the DTI1 and DTI2 measurement points. DTI1’s one-way ANOVA [*F*(2,24) = 6.849, *p* < 0.001] revealed a significant effect between COC-SAL and SAL-SAL groups (*n* = 8–9, **p < 0.01, Bonferroni’s multiple comparison test). No significant effect was revealed between the three groups in the DTI2 point.

For the benefit of clinical use, we asked if the above changes in proliferation and neurogenesis are evident using a non-invasive method—DTI. This method provides a broader picture of the brain’s physiological alterations, including tissue density and organization (Alexander et al., 2007). We conducted another self-administration study, repeating the same schedule as in the previous experiments. Then, rats were MRI scanned for DTI acquisition according to a schedule consistent with the measurement of proliferation and neurogenesis (DTI1 and DTI2; Figure 3A).

The results show a significant effect of DHEA treatment on tissue directionality and organization in the DTI2 FA parameter (Supplementary Figure 2B) between the COC-SAL and COC-DHEA groups). In the DTI1 ADC parameter, one-way ANOVA revealed significantly higher diffusion in the SAL-SAL group vs. the COC-SAL group (Figure 3C) indicating lower tissue density. No significant main effect was found between the SAL-SAL and COC-DHEA groups.

This result creates a paradox. On the one hand, the DTI scan demonstrated an increase in tissue density in the DG in the COC-SAL group, i.e., more cells, suggesting an increase in neurogenesis. On the other hand, our cell number measurement in the previous experiments showed fewer cells, suggesting a decrease in neurogenesis. To understand these contradicting suggestions of the findings, we focused on a significant factor that affects tissue density: astrocytes. Astrocytes have been implicated in the pathophysiology of a number of neuropathological conditions, as they regulate the extracellular space volume and modulate synaptic plasticity. Hence, we investigated the effect of cocaine administration and DHEA treatment on astrocytes in the DG.

### The Effect of Dehydroepiandrosterone Treatment on Astrocyte Cells

**FIGURE 4:**
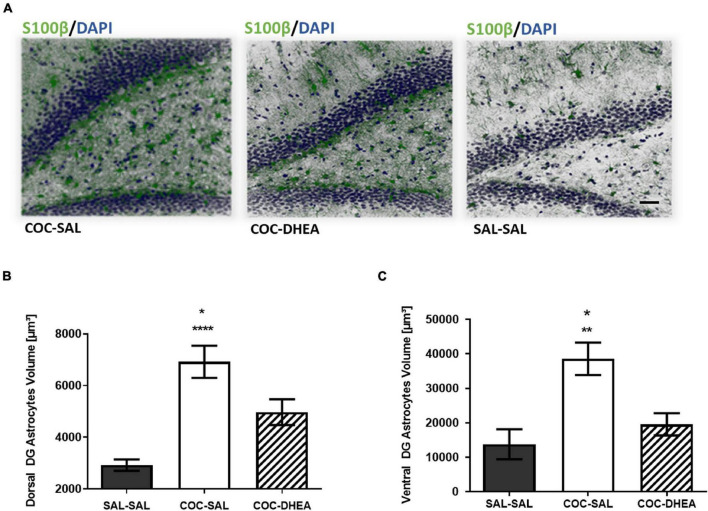
The effect of DHEA treatment on dentate gyrus astrocytes. **(A)** Representative dorsal dentate gyrus images taken under a confocal microscope. Slices were immunohistochemically stained for s100β protein (green) and DAPI (blue). **(B,C)** Quantification of S100β expression in the dorsal and ventral dentate gyrus. One-way ANOVA of S100β expression in all three groups revealed a significant effect between COC-DHEA vs. COC-SAL (*p < 0.05) and SAL-SAL vs. COC-SAL (****p < 0.0001) groups in the dorsal dentate gyrus [*F*(2,13) = 19.99; *p* < 0.001] and between COC-DHEA vs. COC-SAL (*p < 0.05) and SAL-SAL vs. COC-SAL (**p < 0.01) groups in the ventral dentate gyrus [*F*(2,15) = 9.780; *p* < 0.01] (*n* = 5–6, * Bonferroni’s multiple comparison test).

Since astrocytes, the most abundant glial cells in the brain, tend to swell following withdrawal from repeated cocaine administration (Bowers and Kalivas, 2003), we evaluated the total expression of S100β protein, a mature astrocyte marker (Raponi et al., 2007), in the DG in correspondence to the DTI1 scan. The results show a significant increase in S100β expression after repeated cocaine administration, comparing the SAL-SAL and COC-SAL groups. Notably, DHEA treatment has a beneficial effect in reducing S100β expression, comparing COC-DHEA and COC-SAL groups (Figures 4A,B). Interestingly, we also found differences in the ADC parameter in the ventral DG (Figure 3B), which is responsible for emotional memory. We investigated this area, too, since emotional memory is associated with addiction (Goldfarb and Sinha, 2018). One-way ANOVA revealed a significant difference between the COC-DHEA and COC-SAL groups in S100β expression in the ventral DG (Figure 4C). Remarkably, the beneficial effect of DHEA treatment in reducing S100β expression is shown in spacial (DG) and emotional (ventral DG) memory areas, both connected with addiction.

## Discussion

This study aimed to examine the long-term beneficial effect of DHEA treatment on cocaine addiction. Our previous studies demonstrated that DHEA treatment significantly reduces cocaine-seeking behavior. Our current findings shows that this effect endures at least a month after treatment and is due/through normalizing the neurogenesis levels in the dorsal DG. We also show that neurogenesis was correlated with the DTI FA parameter, which determines the axons’ integration and organization (Friedrich et al., 2020). The FA parameter showed a correction in the axon integration in the long-term (a month) after DHEA treatment. The isotropies (FA) declined with time due to the interruption of astrocytes to the axon’s integrity (Toy and Namgung, 2013).

The ADC parameter, which examines the average diffusion (Harkins et al., 2009), showed an increase in tissue density, in the dorsal DG, following withdrawal from repeated cocaine administration in the short term despite the immediate decrease in the number of cells measured in the proliferation. We note that our explanation of low density, presented by ADC parameter, relates to astrocytes since they are the main factor in the brain parenchyma affecting the extracellular space. However, other factors may also be involved in this interruption (Cunningham et al., 2019).

Since DTI is not invasive, these measures can be translated to the clinics for a mirror evaluation of the rehabilitation progress.

We demonstrated, by evaluation of S100β protein expression, a mature astrocyte marker that is highly expressed in protoplasmic astrocytes in rodents, that the increase in the tissue density results, apparently, from increased astrocyte cell volume caused by the drug withdrawal effect (Scofield et al., 2016) rather than from cell proliferation and neurogenesis. The DHEA treatment reduced S100β protein expression, e.g., astrocyte cell volume in the short term, and allowed newborn neurons maturation (neurogenesis) and connectivity (DTI FA parameter).

At the beginning of drug abstinence, metabolic distress affects the astrocytes’ morphology and activity, resulting in reactive astrogliosis that can eliminate neuronal axonal growth (Neal and Richardson, 2018) and impair the neurogenesis process. DHEA is a neuroactive steroid with neuroprotective effects demonstrated in various experimental models regarding astrocytes and neurons. Furthermore, DHEA applies neuroprotective actions through the modulation of water balance by decreasing primary water channel expression, reactive astrogliosis, and several astrocyte functions, including glutamate regulation (Arbo et al., 2016). There is evidence that DHEA increases the production of neurotrophins, such as BDNF and nerve growth factor (NGF) (Rahmani et al., 2013), and neurogenesis in the dentate gyrus of mice with damaged neurogenesis in the subgranular zone (Moriguchi et al., 2013). Our findings are in line with these studies, and further show that DHEA treatment operates on both levels: astrocytes stabilization and neurogenesis.

The current study results emphasize that DHEA proves its effectiveness *via* onset of the neurogenesis progress. Accordingly, DHEA affects the intactness of the neurogenesis process by moderating astroglial reactivity, which is a prerequisite to complete neurogenesis connectivity. Therefore, we suggest that DHEA bi-level effects are associated with drug extinction (washout, detoxification). This agrees with Becker’s theory, according to which newly formed neurons support adaption to changed environmental circumstances, and as such, the flexibility of new neurons to merge into new properties is raised by the microenvironment (Becker, 2005). Otherwise, damaged neurogenesis may lead to impaired adaptation, which is essential to rehabilitation from drug addiction.

To conclude, DHEA can rescue brain damage following exposure to substance intake. Such may serve as a treatment that corrects integrity during abstinence from the substance and not just a relief from withdrawal symptoms as drug replacement treatments offer (Tomkins and Sellers, 2001). Elucidating the propagation of the immediate response of astrocytes at stressful drug abstinence may help understand the development of addiction diseases. Therapies designed to improve astrocyte function after exposure to substances of abuse may represent a unique strategy for the protecting neurogenesis and enhancing brain recovery by defining a specific window of opportunity for intervention to improve rehabilitation.

## Data Availability Statement

The raw data supporting the conclusions of this article will be made available by the authors, without undue reservation.

## Ethics Statement

The animal study was reviewed and approved by Institutional Animal Care and Use Committee (IACUC), Bar-Ilan University Ramat-Gan 52900.

## Author Contributions

HA-L contributed to behavioral, histological experiments and data analyses, and wrote the manuscript. OC and ES participated in the former design of the experiments and critically assisted with different aspects of the behavioral experiments. TB and HP-N assisted with the behavioral experiments. IG contributed to the surgical procedures and animal care and rehabilitation. AJ assisted with the microscopy data analyses. RM and AW provided critical interpretations revision of the manuscript. GY contributed to the research concept and design, interpretation of the results, and critical revision of the manuscript. All authors reviewed the content and approved the final version for publication.

## Conflict of Interest

The authors declare that the research was conducted in the absence of any commercial or financial relationships that could be construed as a potential conflict of interest.

## Publisher’s Note

All claims expressed in this article are solely those of the authors and do not necessarily represent those of their affiliated organizations, or those of the publisher, the editors and the reviewers. Any product that may be evaluated in this article, or claim that may be made by its manufacturer, is not guaranteed or endorsed by the publisher.
