# Supplementary material for: The Effect of Dehydroepiandrosterone Treatment on Neurogenesis, Astrogliosis and Long-Term Cocaine-Seeking Behavior in a Cocaine Self-Administration Model in Rats
Source: Front Neurosci. 2021 Nov 26;15:773197. doi: 10.3389/fnins.2021.773197 (PMC8662380; doi:10.3389/fnins.2021.773197)
Supplement: Supplementary file 1 [file Image_1.pdf]

Supplementary figures and legends

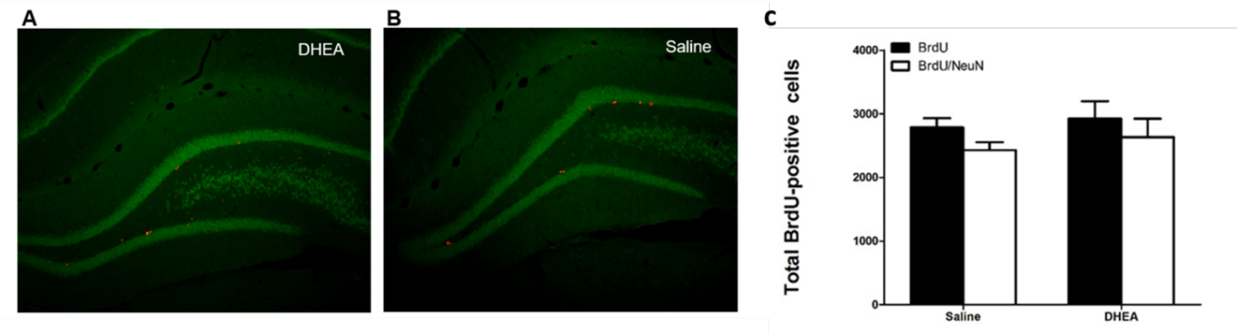

S1. The effect of chronic DHEA treatment on dorsal dentate gyrus neurogenesis in naive rats. A) Representative micrograph (double labeling) of newly generated neurons NeuN+ (green) and BrdU positive (red) in naïve rats treated with saline (A) or DHEA (B). C) Quantification of BrdU positive and NeuN positive cells in the dentate gyrus of naïve rats injected with DHEA or saline. One-way ANOVA of the number of newly formed cells and neurons revealed no significant effect.

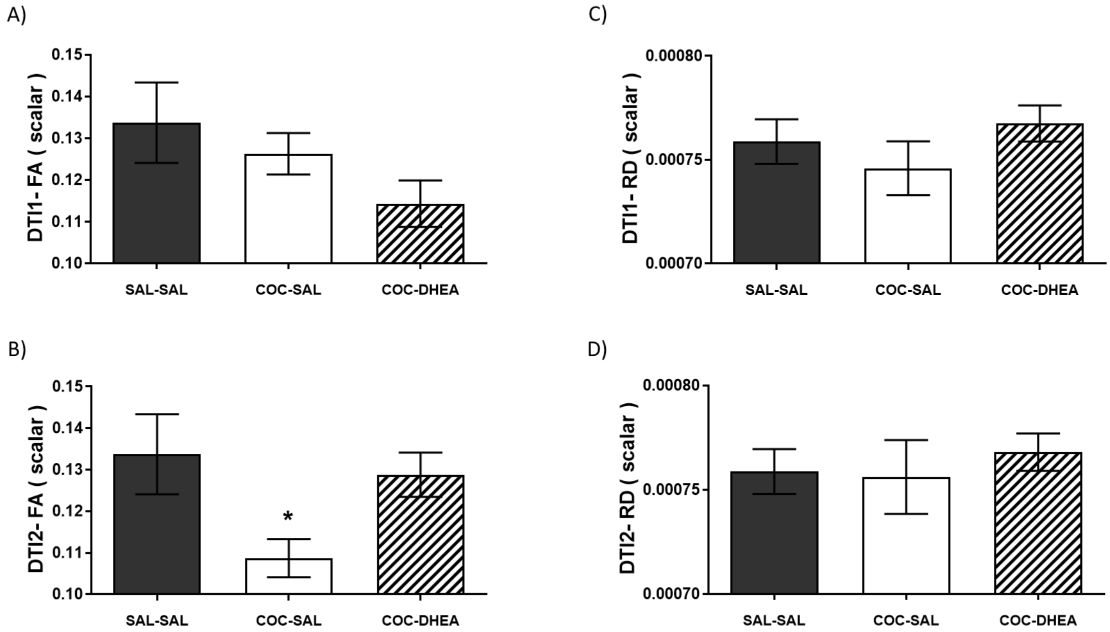

S2. Effect of DHEA treatment on DTI fractional anisotropy and radial diffusivity parameters of dentate gyrus area after cocaine self-administration. A-B) Quantification of motional anisotropy (FA) of water molecules in DTI1 and DTI2 points, respectively. In DTI1, one-way ANOVA revealed no significant effect. In the DTI2 point, COC-SAL vs. SAL-SAL groups showed a significant main effect (* $t=2.776$, $df=16$; $p<0.05$). Diversely**,** COC-DHEA vs. SAL-SAL groups showed no significant main effect**.** C-D) Quantification of averaged diffusivities in the two minor axes (RD) in the DTI1 and DTI2 measurement points; one-way ANOVA revealed no significant effect.
